# An intelligent diagnosis method for cardiovascular diseases based on the CNN-CBAM-GRU model

**DOI:** 10.1371/journal.pone.0330279

**Published:** 2025-09-02

**Authors:** Zheng Gong, Yufeng Chen, Shirong Lin, Jun Ke, Juying Huang, Hongyi Chen, Hongyu Huang, Yue Shen, Yi Gu, Lixun Chen, Feng Chen

**Affiliations:** 1 Shengli Clinical Medical College of Fujian Medical University, Fujian Medical University, Fuzhou, Fujian, China; 2 Department of Emergency, Fujian Provincial Hospital, Fuzhou, Fujian, China; 3 Fuzhou University Affiliated Provincial Hospital, Fuzhou, Fujian, China; 4 Fujian Provincial Key Laboratory of Emergency Medicine, Fuzhou, Fujian, China; 5 The Outpatient Department of Fujian Provincial Hospital, Fuzhou, Fujian, China; 6 School of Informatics, Xiamen University, Xiamen, Fujian, China; 7 School of Artificial Intelligence and Computer Science, Jiangnan University, Wuxi, China; Khalifa University of Science and Technology, UNITED ARAB EMIRATES

## Abstract

Early diagnosis of cardiovascular diseases (CVDs) is essential for improving patient outcomes. As a primary diagnostic modality, electrocardiogram (ECG) signals pose challenges for automatic classification due to their complex temporal and morphological characteristics. This study proposes a CNN-CBAM-GRU model that integrates Convolutional Neural Networks (CNN), the Convolutional Block Attention Module (CBAM), and Gated Recurrent Units (GRU) to enhance both spatial feature representation and temporal sequence modeling. The model is evaluated on two public ECG datasets—MIT-BIH and PTB-XL—under five-class classification settings. Unlike many existing approaches that report only a limited set of metrics, this study conducts a comprehensive evaluation across multiple performance indicators, including accuracy, precision, recall, sensitivity, and F1-score, providing a more complete view of classification effectiveness. Experimental results demonstrate that the proposed model achieves a strong balance between predictive performance and computational efficiency. Specifically, it achieves 98.17% accuracy and 98.91% F1-score on MIT-BIH, and 99.21% accuracy and 99.47% F1-score on PTB-XL, with a compact parameter size of 2.45 million. These findings validate the proposed model as a practical and robust solution for intelligent ECG classification and automated cardiovascular disease diagnosis.

## 1. Introduction

Cardiovascular diseases (CVDs) are a leading cause of mortality worldwide, affecting individuals of all age groups [1]. According to the World Health Organization (WHO), CVDs result in nearly 18 million deaths annually, accounting for approximately 31% of global fatalities. These diseases, including coronary heart disease, stroke, and hypertension, pose a significant threat to both life expectancy and quality of life [2]. Traditional diagnostic methods for CVDs, such as history taking, physical examinations, ECGs, echocardiograms, and blood tests, help assess heart function and identify abnormalities like arrhythmias and myocardial ischemia. Among these, the ECG is a non-invasive, real-time tool that provides vital information about the heart’s electrical activity and is essential for early detection and urgent evaluations of CVDs [3].

In recent years, significant progress has been made in the use of artificial intelligence (AI) and machine learning (ML) for the early diagnosis of cardiovascular diseases. AI techniques have revolutionized ECG analysis, allowing for more accurate and efficient identification of heart conditions. For instance, Esina et al. (2020) developed a method using ECG scattering mapping with 80% sensitivity and 70.8% specificity, but it struggled with identifying risk factors [4]. Karboub et al. (2021) employed SVM, CNN, and other methods combined with CWT and DWT feature extraction to achieve 99.92% accuracy, though at the cost of high computational complexity [5]. Other models, like those by Cheng et al. (2021), used CNN and BiLSTM to enhance classification performance, but heavily relied on labeled data [6]. More recent models, such as the DLECG-CVD model by Karthik et al. (2022), showed excellent accuracy but faced challenges with complex training processes [7].

In addition to traditional deep learning frameworks, several recent models have attempted to address the limitations of ECG classification. For example, Guan et al. [8] proposed the HA-ResNet model, which leverages hidden attention mechanisms and two-dimensional signal transformation to enhance arrhythmia detection performance. However, its high computational complexity limits its deployment in real-time applications. Tayyeb et al. [9] implemented a lightweight MLP-based classifier with 94.40% accuracy, though it underperformed in multi-class or ambiguous cases. Jyotishi et al. [10] developed ASTLNet, a spatio-temporal fusion network for multi-lead ECG analysis, achieving high accuracy but at the cost of increased model size and training time. Wajgi et al. [11] proposed an enhanced lemurs optimization-based hybrid attention network for ECG arrhythmia classification, which employs optimal weighted feature selection to improve accuracy and robustness; however, it still incurs high computational cost due to its complex optimization structure.

Despite the promising advances, several challenges persist in deep learning-based ECG analysis. First, many existing models struggle with effective feature extraction, particularly in capturing critical temporal features from sequential ECG data, which may hinder classification performance [12]. Second, conventional CNN architectures often lack explicit modeling of inter-channel relationships, leading to suboptimal representation learning [13]. Third, although some models achieve high accuracy, their high computational complexity limits their applicability in real-time or resource-constrained clinical environments [14].

To address these issues, this paper proposes a novel lightweight architecture named CNN-CBAM-GRU, which integrates a Convolutional Block Attention Module (CBAM) into a CNN-GRU framework. CBAM enables the model to adaptively refine spatial and channel-wise features by focusing on the most informative components, thereby enhancing the overall feature extraction capability.

The main contributions of this study are summarized as follows:

(1) A novel CNN-CBAM-GRU architecture is proposed, where the CBAM module is embedded within a CNN-GRU framework to improve the model’s attention to key spatial and temporal features in ECG signals.
(2) The combination of 1D convolutional layers with CBAM enhances spatial feature representation, while the GRU layer captures temporal dependencies, enabling more accurate classification of diverse cardiac conditions.
(3) Experimental results on benchmark ECG datasets demonstrate that the proposed model achieves superior performance compared to conventional CNN-GRU models, maintaining a balance between classification accuracy and computational efficiency, and thus showing strong potential for real-world cardiovascular disease diagnosis.

The rest of this paper is organized as follows. Section 2 reviews related work, and Section 3 introduces the proposed method. Section 4 presents the experimental results and analysis. Finally, Section 5 concludes the paper.

## 2. Previous works

There are many different diagnostic methods for cardiovascular diseases, each with its own characteristics. Among them, the ECG-based diagnostic method has attracted much attention due to its real-time and non-invasive nature [15]. Compared with other imaging or biochemical methods, ECG has significant advantages such as high portability, low cost, and suitability for long-term monitoring. The ECG-based cardiovascular diagnostic process includes signal acquisition, preprocessing, classification (based on prior knowledge or deep learning methods) and classification result generation, and finally outputs the diagnostic result.

At present, ECG signal analysis based on deep learning technology has made significant progress in arrhythmia detection. Niu et al. [16] proposed a deep learning method based on adversarial domain adaptation to cope with data distribution differences and improve cross-domain classification performance. Hua et al. [17] used a one-dimensional convolutional neural network (1D-CNN) for time series feature extraction, which effectively improved the efficiency of ECG classification, but still had limitations when processing complex signals. Golrizkhatami and Acan [18] significantly enhanced the feature extraction capability by fusing three-layer feature descriptors, providing a new idea for multi-Class arrhythmia classification.

In addition, Oh et al. [19] proposed a model combining CNN and LSTM technology for variable-length heartbeat signal classification, and optimized the model performance by combining local feature extraction with long-term dependency modeling. The CNN-BLSTM network-based system developed by Shoughi and Dowlatshahi [20] performed well on unbalanced datasets, demonstrating its potential in dealing with data imbalance problems. To further optimize the model performance, Chen et al. [21] proposed a model combining 1D_CBAM autoencoder and lightweight CNN, providing an effective solution for resource-constrained scenarios. Singh and Mahapatra [22] used recursive CNN combined with grey wolf optimization (GWO) to demonstrate efficient performance in real-time diagnosis tasks.

Although the above studies have made significant progress in ECG-based cardiovascular disease diagnosis, there are still some challenges: first, the limitation of feature extraction. Traditional deep learning models may not be able to fully capture the important temporal features in ECG signals; second, the neglect of feature channel relationships. The channel dependencies in deep learning models are usually implicit and local; finally, computational cost and efficiency issues still affect the feasibility of practical applications.

Based on this, recent studies have begun to explore solutions to the above problems by introducing attention mechanisms. For example, the Lead-fusion Barlow Twins method achieves efficient and robust arrhythmia detection with limited labeled data by combining a self-supervised learning model with multi-lead information. The Adversarial Spatiotemporal Contrastive Learning (ASTCL) method [23] demonstrates excellent performance in multi-Class classification tasks by adversarial modeling of time series and spatial distribution features. Attention-based Time-Incremental Convolutional Neural Network (ATi-CNN) [24] effectively processes 12-lead ECG signals of different lengths, while Transformer-based deep neural networks [25] extract important features from continuous ECG signals by modeling long-distance dependencies, and perform well in arrhythmia classification. In addition, the ECG encode method [26] proposes a compact and computationally efficient feature encoding method to achieve high-quality feature extraction under resource-constrained conditions. These methods have promoted the advancement of ECG-based cardiovascular diagnosis technology to varying degrees, but there is still a need to further balance the complexity of the model with the needs of practical applications.

## 3. Method

### 3.1 Network architecture

**Fig 1:**
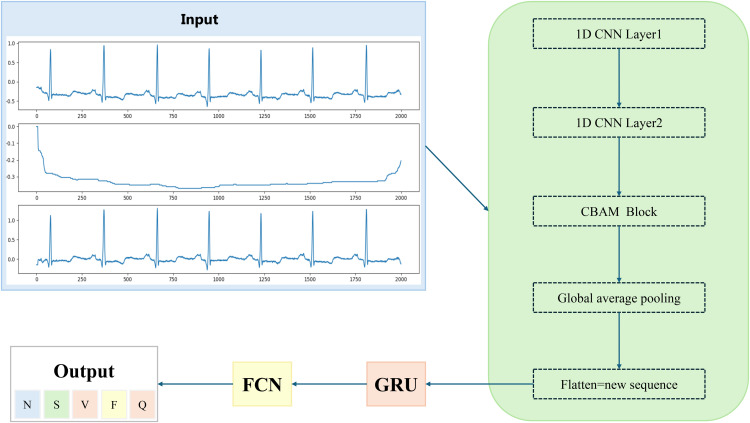
CNN-CBAM-GRU network structure.

This study proposed a network architecture based on CNN-CBAM-GRU, which embeds the CBAM into CNN to improve the feature extraction capability in ECG classification tasks. CBAM assigns dynamic weights to feature channels through the attention mechanism, thereby optimizing the important features in the signal. Then, the enhanced features are modeled in time series through the GRU module, and finally efficient classification is achieved. Experimental results show that CNN-CBAM-GRU outperforms the traditional CNN-GRU model in classification accuracy and overall performance. The proposed CNN-CBAM-GRU network structure is shown in Fig 1.

The model is designed to perform intelligent diagnosis of cardiovascular diseases and identify potential heart diseases by classifying ECG signals. The input is an ECG signal, which first passes through two 1D convolutional layers (1D CNN Layer1 and 1D CNN Layer2) to extract local features from the ECG signal. These features are further processed in the following CBAM. The CBAM dynamically adjusts the weights of important features through channel attention and spatial attention mechanisms, thereby enhancing the network’s attention to key information. Then, the features are reduced in dimension through the Global Average Pooling operation, and then the pooled features are flattened and input into the GRU layer. The GRU is used to capture the timing features in the ECG signal and further extract the temporal dependency. Finally, the output of the GRU is classified through a fully connected layer to output the diagnosis results of cardiovascular diseases. The entire network structure uses deep learning technology, combined with the advantages of convolution and recurrent neural networks, to effectively process the spatiotemporal features of ECG signals and provide accurate disease diagnosis results.

### 3.2. CBAM

**Fig 2:**
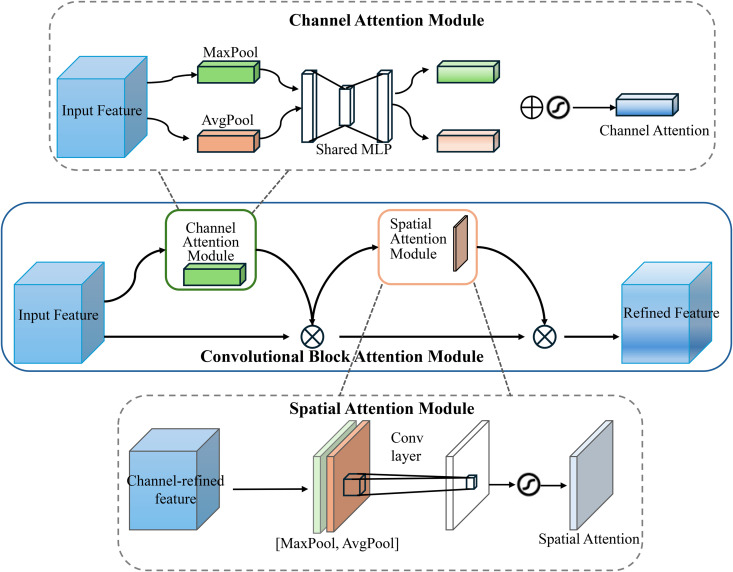
CBAM structure.

CBAM is an attention mechanism [27] that combines information from both spatial and channel dimensions to implement attention, serving as a complement to SE [28]. By introducing channel attention and spatial attention modules, CBAM effectively enhances the feature representation ability of convolutional neural networks. The channel attention module generates weights for each channel by performing max pooling and average pooling operations on the feature map, emphasizing the important channel information. The spatial attention module, on the other hand, generates weights for spatial positions by pooling across the channel dimension, helping the network focus on key regions. These two attention mechanisms can be applied sequentially, adjusting the channel and spatial information of the input feature map through weighted operations, ultimately improving the network’s performance across various computer vision tasks. The structure of CBAM is shown in Fig 2, adapted from Ref. [29]. CBAM has a simple structure with low computational overhead, and can seamlessly integrate into existing convolutional network architectures to enhance their attention to important features [30].

The calculation process of CBAM is as follows:

(1) Let the input feature map $X\in R^{H\times W\times C}$, where *H* is the height, *W* is the width, and *C* is the number of channels.
(2) The goal of the channel attention module is to adjust the feature map based on the importance of each channel. The specific steps are as follows:

First, perform global average pooling $G_{avg}(X)\in R^{1\times 1\times C}$ as defined in Eq. (1), and global max pooling $G_{max}(X)\in R^{1\times 1\times C}$ as defined in Eq. (2), on the input feature map:


$$G_{avg}(X)=\frac{1}{H\times W}\sum {\mathrm{\backslash nolimits}}_{i=1}^{H}\sum {\mathrm{\backslash nolimits}}_{j=1}^{W}X(i,j,k)$$ (1)

$$G_{max}(X)=\underset{i,j}{\text{max}}X(i,j,k)$$ (2)

Where *i* and *j* are spatial dimensions, and *k* is the channel index.

Then, process the pooled features through shared fully connected layers (typically two fully connected layers with ReLU activation). The specific operations are given in Eq. (3) and Eq. (4):


$$F_{avg}(X)=\sigma (W_{2}\cdot ReLU(W_{1}\cdot G_{avg}(X)))$$ (3)

$$F_{max}(X)=\sigma (W_{2}\cdot ReLU(W_{1}\cdot G_{max}(X)))$$ (4)

Where $W_{1}$ and $W_{2}\;$ are the weight matrices of the fully connected layers, and σ is the Sigmoid activation function. The generated $F_{avg}(X)$ and $F_{max}(X)$ are the channel attention feature maps.

Finally, the channel attention map is computed by summing the above two results and applying the Sigmoid function, as shown in Eq. (5):


$$M_{c}(X)=\sigma (F_{avg}(X)+F_{max}(X))$$ (5)

The final output is then obtained by element-wise multiplication of the original input feature map with the channel attention map, as given in Eq. (6):


$$X^{\prime }=X\cdot M_{C}(X)$$ (6)

(3) The goal of the spatial attention module is to adjust the feature map based on the importance of spatial positions. The processing flow is as follows:

First, perform channel aggregation on the input feature map *X*’ (which has already undergone channel attention adjustment).Compute the max and average values for each spatial position across the channels, as shown in Eq. (7) and Eq. (8):


$$M_{avg}\left(X^{\prime }\right)=\frac{1}{C}\sum {\mathrm{\backslash nolimits}}_{c=1}^{C}X^{\prime }(i,j,c)\;\;\;\;\;\;\;\;\;\;\;\;\;\;\;\;\;\;$$ (7)

$$M_{max}\left(X^{\prime }\right)=\underset{c}{\text{max}}X^{\prime }(i,j,c)$$ (8)

These two results are *H* x *W* spatial feature maps.

Then, concatenate the above two spatial feature maps and process them through a 7x7 convolution layer to obtain the spatial attention map, as shown in Eq. (9):


$$M_{s}\left(X^{\prime }\right)=\sigma ({Conv}_{7\times 7}(M_{avg}(X^{\prime })||M_{max}(X^{\prime })))$$ (9)

Where || denotes the concatenation operation, and Conv is the convolution operation, generating the spatial attention map $M_{s}(X^{\prime })$.

(4) The final output feature map *X*“ is the feature map that has undergone both channel and spatial attention adjustments. It automatically highlights important features while suppressing irrelevant ones, thereby improving the model’s representation ability and performance [31,32].

## 4. Experimental results and analysis

### 4.1. Dataset

To comprehensively evaluate the performance of the proposed model in ECG classification tasks, this study employs two authoritative public databases: the MIT-BIH Arrhythmia Database [33] and the PTB-XL Dataset [34]. The MIT-BIH Arrhythmia Database contains 48 half-hour ECG recordings from 47 subjects, sampled at 360 Hz. Based on the AAMI EC57 standard, heartbeats are categorized into five classes: Normal (N), Supraventricular ectopic (S), Ventricular ectopic (V), Fusion beats (F), and Unclassified beats (Q). After preprocessing and segmentation into 10-second intervals, the original dataset yielded approximately 87,554 labeled samples: N (75,005), S (3,724), V (6,723), F (803), and Q (1,299). The class distribution is evidently imbalanced, with the Normal class dominating the dataset. The PTB-XL Dataset, provided by PhysioNet, consists of 21,837 12-lead ECG records collected at a sampling rate of 500 Hz. According to its official diagnostic annotations, five diagnostic superclasses are defined: Myocardial Infarction (MI), Conduction Disturbance (CD), Hypertrophy (HYP), ST/T Change (STTC), and Normal ECG (NORM). After filtering and segmenting into 10-second samples, this study retained 104,146 labeled segments for classification: NORM (78,050), STTC (7,485), MI (10,992), CD (6,912), and HYP (711), again exhibiting noticeable class imbalance.

To ensure consistency in evaluation across datasets, both MIT-BIH and PTB-XL were independently processed for 5-class classification based on their respective labeling schemes. A uniform preprocessing pipeline was applied: (1) A 0.5–50 Hz band-pass filter was used to eliminate baseline drift and high-frequency noise. (2) All signals were normalized to zero mean and unit variance. (3) ECG signals were segmented into non-overlapping 10-second intervals, preserving the original time sequence.

Given the strong class imbalance observed in both datasets, the Synthetic Minority Oversampling Technique (SMOTE) was applied to the training set to balance class distribution. This approach avoids simple duplication and instead synthetically generates new samples for minority classes, improving the model’s capacity to learn from underrepresented patterns and reducing overfitting risks. The final distribution of the datasets after segmentation and SMOTE-based balancing is summarized in Table 1.

**Table 1:** Detailed division of the preprocessed data set.

Dataset	Class	Total	Training set	Validation set	Test set
MIT-BIH	N	90,000	72,000	9,000	9,000
	S	80,000	64,000	8,000	8,000
	V	1,0000	8,000	1,000	1,000
	F	1,0000	8,000	1,000	1,000
	Q	1,0000	8,000	1,000	1,000
PTB-XL	N	1,0000	8,000	1,000	1,000
	STTC	1,0000	8,000	1,000	1,000
	MI	1,0000	8,000	1,000	1,000
	CD	1,0000	8,000	1,000	1,000
	HYP	1,0000	8,000	1,000	1,000

### 4.2. Experimental parameter settings

As can be seen in Table 2, the CNN-CBAM-GRU model that has been suggested has the following parameter settings, where the parameters of each CNN layer are the same.

**Table 2:** Model parameter settings.

CNN	CBAM	GRU	Fully connected layer	Training Hyperparameters
Kernel filter:32 kernel size: 3 Stride: 1	Channel Attention:Avg & Max Pooling Spatial Attention:7x7 Conv	GRU Units: 64 Layers: 2	FC Units: 128 Activation: ReLU	Learning rate: 0.001 Optimizer: Adam Batch size: 64 Epochs: 200

The experimental context for this study is as follows: The hardware configuration used includes an Intel Core i7 processor, an NVIDIA GTX 1660 graphics card, 16GB of DDR4 memory, and 512GB of SSD storage. For software, Ubuntu 20.04 was used as the operating system, Python 3.8 as the programming language, PyTorch 1.8 as the deep learning framework, and CUDA 11.0 along with cuDNN 8.2 were installed to support GPU acceleration. The following are the experimental evaluation indicators:


$$Accuracy=\frac{TP+TN}{TP+FP+TN+FN}$$ (10)

$$\mathrm{Pr}ecision=\frac{TP}{TP+FP}$$ (11)

$$Re\mathrm{\backslash nolimits}call=\frac{TP}{TP+FN}$$ (12)

$$Sen=\frac{TP}{TP+FN}$$ (13)

$$F1=\frac{2*Re\mathrm{\backslash nolimits}call*\mathrm{Pr}ecision}{Re\mathrm{\backslash nolimits}call+\mathrm{Pr}ecision}$$ (14)

Equation (10) through Equation (14) define the evaluation metrics used in this study for cardiovascular disease detection. In the evaluation of cardiovascular disease detection, the following metrics are defined: True Positive (TP) refers to instances where heart rate readings are correctly identified as indicative of cardiovascular disease. True Negative (TN) represents cases where heart rate readings are accurately identified as not indicative of cardiovascular disease. False Positive (FP) denotes instances where heart rate readings are incorrectly classified as indicative of cardiovascular disease when they are not. False Negative (FN) indicates cases where heart rate readings are incorrectly classified as not indicative of cardiovascular disease when they are.

### 4.3. Hyperparameter optimization

To determine the optimal set of hyperparameters for the model, a series of tuning experiments were conducted exclusively on the training and validation sets. The key parameters explored include the learning rate, batch size, number of convolutional filters, kernel size, and the number of GRU units. For each configuration, the model was trained on the training set and its performance was evaluated on the validation set. Table 3 summarizes the classification accuracy on the validation set under different hyperparameter combinations in the 5-class classification task. To ensure fair evaluation, all experiments were conducted using the balanced datasets generated via the SMOTE algorithm, where each class in the training set had an equal number of samples to mitigate class imbalance and improve generalization.

**Table 3:** Model performance under different parameter settings.

No.	Learning Rate	Batch Size	Num Filters	Kernel Size	GRU Units	Accuracy(%)
1	0.001	32	16	3	32	96.33
2	**0.001**	**64**	**16**	**3**	**64**	**98.17**
3	0.01	64	32	3	32	97.68
4	0.01	64	32	5	64	97.90
5	0.001	32	32	5	64	96.92
6	0.01	32	16	3	64	96.80

According to the results presented in Table 3, the highest classification accuracy of 98.17% was achieved when the learning rate was set to 0.001, batch size to 64, number of convolutional filters to 16, kernel size to 3, and the number of GRU units to 64. This combination contributes to more stable convergence and improved classification performance.

**Fig 3:**
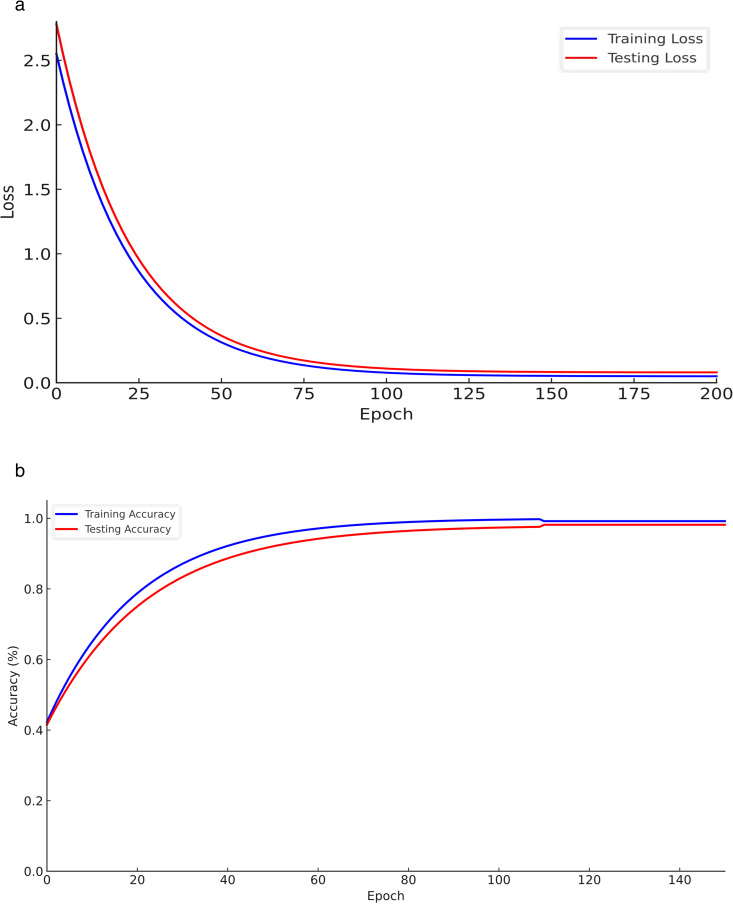
Performance comparison of training set and test set.

To further validate this configuration, Fig 3 illustrates the model’s training and testing performance across epochs. As shown, the loss curves converge smoothly, and the accuracy trends stabilize after approximately 110 epochs, indicating effective learning and strong generalization capabilities.

Both the training and test sets’ loss function values trend with Epoch, as shown in Fig 3(a). It is clear that the loss function value is reducing and tends to minimize the error as the model parameters are regularly updated. Typically, the shift becomes steady around Epoch 100, when the training set’s loss function value hits its lowest point. The test set’s loss function value hits rock bottom at approximately 110 Epoch and then stays rather constant after that. The model has converged throughout testing and training, as shown above. Observing the trend of the loss function value reveals that the model is capable of successfully reducing error during training and testing and that the loss function value remains stable at a low level. This indicates that the model has converged and learned all of the characteristics from the data.

Accuracy trends on both the training and test sets are displayed in Fig 3(b). Although the two sets of data show almost identical patterns of change, the training set typically has superior accuracy. The precision keeps getting better as the Epoch gets higher. Training set accuracy peaks at around 100 Epoch and then tends to stabilize; test set accuracy peaks at around 110 Epoch and then tends to stabilize as well. After reaching its maximum value, the model’s accuracy tends to settle, and it shows substantial improvement in performance on both the training and test sets. This demonstrates that the model performs admirably on test data as well as training data when it comes to generalization. The model can adapt to the data and improve accuracy throughout training, even when the training set has better accuracy than the test set. The fact that both sets show a constant improvement trend further supports this claim. As a whole, the model handled the data and predictions adequately during the experiment.

### 4.4. Comparison of model performance and model design insights

#### 4.4.1 Quantitative performance comparison.

To comprehensively evaluate the performance differences of various models in cardiovascular disease classification tasks, this study compares multiple existing methods based on their experimental results on the same datasets (MIT-BIH and PTB-XL). Tables 4 and 5 present the experimental results of each model across multiple metrics in the 5-class classification task. All comparison results are based on the experimental settings and performance reported in the original literature, ensuring fairness and consistency in the evaluation. To ensure consistency in comparison, and given that existing methods generally report only average performance metrics, this study also adopts the average value as the primary evaluation result. All reported results are the mean of three independent runs of the model.

**Table 4:** 5-Class classification results of related studies on the MIT-BIH dataset.

Method	Accuracy	Precision	Recall	Sen	F1	Param.(M)
Hua et al. [17]	97.00	–	–	97.00	97.00	–
Golrizkhatami et al [18]	98.00	–	–	93.57	–	–
Oh et al. [19]	98.1	–	–	97.5	–	–
Shoughi et al. [20]	99.48	–	–	94.44	–	–
Chen et al. [21]	98.21	–	–	95.03	96.24	1.28
Singh et al. [22]	98.2	–	–	–	–	–
Bayani et al. [35]	99.38	99.60	99.40	–	99.60	–
Proposed Method	98.17	98.54	99.29	97.86	98.91	2.45

**Table 5:** 5-Class classification results of related studies on the PTB-XL dataset.

Method	Accuracy	Precision	Recall	Sen	F1	Param.(M)
Kancharla et al. [36]	97.0	97.3	96/8	99.5	–	–
Bhanjaa et al. [37]	97.5	97.62	97.43	–	97.52	–
Śmigiel et al. [38]	76.50	71.40	66.20	–	68.00	0.58
Bayani et al. [35]	99.24	99.09	99.02	–	99.05	–
Proposed Method	99.21	98.96	99.98	98.95	99.47	2.45

As presented in Table 4, the proposed CNN-CBAM-GRU model achieves an accuracy of 98.17% on the MIT-BIH dataset, along with high values in precision (98.54%), recall (99.29%), sensitivity (97.86%), and F1-score (98.91%). Compared to previous studies, most of which only reported accuracy or partial metrics, the proposed model provides a more comprehensive performance profile across multiple evaluation dimensions. For instance, while methods such as Shoughi et al. [20] and Bayani et al. [35] achieved higher accuracy values (99.48% and 99.38%, respectively), they lack detailed reporting on sensitivity, F1-score, or model complexity, making it difficult to assess their reliability in imbalanced class scenarios or real-time deployment. In contrast, the proposed model achieves competitive accuracy while maintaining a relatively lightweight structure of 2.45M parameters and ensuring robust classification across all heartbeat categories. Additionally, the model demonstrates strong recall and sensitivity, particularly important for recognizing minority classes such as F and Q, which often have low representation in ECG datasets. This highlights the model’s capacity for handling class imbalance and its suitability for clinical applications requiring reliable identification of rare yet critical cardiac events. Overall, the results indicate that the integration of CBAM and GRU effectively enhances the model’s ability to capture both spatial and temporal dependencies in ECG signals, leading to balanced and stable classification performance across all evaluation metrics.

On the PTB-XL dataset, most methods maintained strong performance in terms of accuracy, but significant differences emerged in model complexity, metric completeness, and minority class recognition capability. The LDCNN model proposed by Bayani et al. [35] achieved the highest reported accuracy (99.24%) and an F1-score of 99.05%, yet did not disclose its parameter size or detailed training configurations, limiting its evaluation in practical deployment scenarios. The lightweight model by Śmigiel et al. [38], with only 0.58M parameters, exhibited advantages in size but suffered from poor classification performance (Accuracy: 76.50%, F1-score: 68.00%), indicating its inability to effectively capture spatiotemporal ECG features. Kancharla et al. [36] combined pretrained CNNs with an XGBoost classifier, obtaining high Precision (97.3%) and Sensitivity (99.5%), but again lacked complete performance metrics, leaving generalization ability uncertain.

In contrast, the proposed CNN-CBAM-GRU model achieves consistently high performance across all key metrics with a parameter size of only 2.45M. It reaches 99.21% Accuracy, 98.96% Precision, 99.98% Recall, 98.95% Sensitivity, and 99.47% F1-score, demonstrating excellent capability in recognizing both dominant and minority ECG classes. The high Recall and F1-score values suggest strong robustness in detecting low-frequency or ambiguous categories, such as F and Q. This superior performance stems from the integration of CBAM for attention refinement and GRU for capturing temporal dependencies, leading to enhanced feature discriminability. Moreover, compared with other high-performing models, the proposed method features a more compact and interpretable architecture, making it well-suited for real-world, scalable deployment in intelligent cardiovascular disease diagnosis systems.

#### 4.4.2 Innovation and advantages of the proposed model.

In recent years, deep learning models combining CNN and GRU have achieved significant progress in various research and application fields. Zhang et al. (2020) proposed a spatio-temporal attention-based convolutional recurrent neural network for multi-class arrhythmia detection [39]. This study demonstrated the effectiveness of attention mechanisms in enhancing feature extraction; however, due to its high complexity, the model’s suitability for lightweight devices is limited. Subsequently, Bhatia et al. (2022) constructed a hybrid deep learning model combining CNN, GRU, and LSTM, which significantly improved ECG signal classification performance [40]. However, this method did not incorporate an attention mechanism to dynamically optimize the importance of feature channels. Ma et al. proposed a multi-class arrhythmia detection method based on ResNet and CBAM, which extracts features using ECG Gramian Angular Summation Field (GASF) augmented by CWGAN-GP, thereby improving classification accuracy. However, the model’s training process relies on the generative adversarial network, which leads to longer training times and higher computational resource consumption [41]. Yadav et al. (2023) proposed a CNN and Bi-GRU architecture that performed well in heartbeat sound classification tasks but lacked an attention mechanism for dynamically adjusting complex features [42]. Zhou and Li proposed an ECG data enhancement method using generative adversarial networks (GANs) combined with Bi-LSTM and CBAM. This method improves the quality of ECG data by generating augmented data with the help of Bi-LSTM and CBAM, which enhances the model’s performance in ECG-related tasks. However, the method relies on the use of GANs, which can result in high computational cost and longer training times [43]. More recently, Chopannejad et al. (2024) developed a hybrid model combining CNN, BiLSTM, and BiGRU, utilizing attention mechanisms to enhance feature extraction for 12-lead ECG signals [44]. However, the complexity of this model makes it challenging to extend to lightweight applications for single-lead ECG devices.

In comparison, the CNN-CBAM-GRU model proposed in this study integrates the CBAM organically into the CNN-GRU architecture. It optimizes the design based on the importance distribution characteristics of ECG signals, enabling CNN to more precisely capture key spatial features while enhancing GRU’s capability for temporal sequence modeling. This model is designed to balance lightweight implementation with broad applicability, effectively adapting to scenarios for single-lead ECG devices. In multi-classification tasks, the CNN-CBAM-GRU model significantly improves classification performance and demonstrates excellent deployment potential, showcasing remarkable innovation and practicality.

#### 4.4.3 Quality validation of SMOTE-generated samples.

To systematically evaluate the quality of ECG samples generated using the SMOTE method, experiments were conducted on the MIT-BIH Arrhythmia Database. The validation focused on two aspects: statistical consistency and spatial distribution visualization, aiming to ensure that the generated samples reflect the morphological diversity of real ECG signals rather than being the result of naive linear interpolation.

Specifically, we extracted the feature representations of each class from the proposed model (using intermediate-layer output vectors), and computed the intra-class variance for both original and SMOTE-generated training samples. The results are presented in Table 6:

**Table 6:** Comparison of intra-class variance between original and SMOTE samples.

Class	Original Variance	SMOTE Variance	Difference (%)
N	0.213	0.227	+6.57%
S	0.185	0.194	+4.86%
V	0.162	0.157	−3.09%
F	0.174	0.179	+2.87%
Q	0.168	0.171	+1.78%

The results demonstrate that the intra-class variance of SMOTE samples closely aligns with that of real samples, with a deviation rate within ±7%. This indicates that the synthetic samples preserve the original distribution characteristics in the feature space.

**Fig 4:**
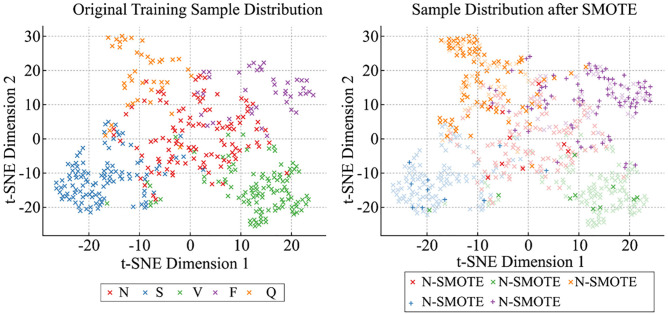
t-SNE visualization of original and SMOTE-augmented ECG samples.

To further examine the spatial structure of the samples, we applied t-distributed Stochastic Neighbor Embedding (t-SNE) to project both real and SMOTE-generated samples into a two-dimensional space. As illustrated in Fig 4, the SMOTE samples are largely embedded within the clusters formed by original samples across all five classes. In particular, for the minority classes (F and Q), the synthetic samples naturally fill the sparse regions without abnormal dispersion or clustering. This observation confirms that SMOTE effectively maintains the class-wise distribution structure during sample generation.

### 4.5 Ablation experiment

To systematically evaluate the effectiveness of the proposed model’s key components and strategies, we conducted a series of ablation studies on the MIT-BIH dataset. These experiments covered various aspects, including data augmentation techniques, attention mechanisms, temporal modeling modules, and the overall architectural composition, aiming to comprehensively assess the contribution of each component to the model’s performance. First, to validate the impact of data augmentation, we compared three different strategies—no augmentation, SMOTE, and WGAN-based augmentation—to examine whether SMOTE significantly improves model performance. The WGAN model employed fully connected generator and discriminator networks and was trained using RMSProp (learning rate = 5 × 10 ⁻ ⁵, batch size = 64) with weight clipping in the range [−0.01, 0.01] and a 5:1 critic-to-generator update ratio. The corresponding results are reported in Table 7. Subsequently, additional ablation experiments were conducted to evaluate the influence of other structural modules. Table 8 presents the performance differences under fixed CNN and GRU settings when adopting different attention mechanisms (none, SE, and CBAM). Table 9 compares various temporal modeling modules under fixed CNN and CBAM configurations. Table 10 summarizes the results of the complete architecture as well as different combinations of modules, providing insights into the effectiveness of the joint design strategy. All experiments were conducted on a five-class classification task using the MIT-BIH dataset, with consistent model architecture and training hyperparameters across all settings. The final performance metrics were obtained by averaging results over three independent runs for each configuration.

**Table 10:** Results of the overall ablation experiment on the model.

CNN	CBAM	GRU	Accuracy	Precision	Recall	F1-score	Param. (M)
√			93.63(90.74,93.48)	93.77(92.45, 94.65)	94.20(93.10, 95.10)	93.98(92.77, 94.87)	1.2
√	√		94.89 (93.65, 95.55)	94.25(93.10, 95.10)	94.97(93.80, 96.00)	95.95(94.79, 96.79)	1.55
√		√	96.88 (95.70, 97.50)	96.96(95.80, 97.60)	97.25(96.10, 98.00)	95.73(94.58, 96.53)	1.8
√	√	√	98.17(97.54,98.96)	98.54(97.55,99.31)	99.29(98.90,99.72)	98.91 (98.22,99.51)	2.45

**Table 7:** Effects of data augmentation on CNN-CBAM-GRU performance.

Methods	Accuracy	Precision	Recall	F1-score
Only CNN-CBAM-GRU	94.06	93.58	92.74	93.15
CNN-CBAM-GRU with SMOTE	98.17	98.54	99.29	98.89
CNN-CBAM-GRU with WGAN	97.28	97.67	98.38	98.22

**Table 8:** Comparison results of attention mechanism ablation.

Metric	No Attention	SE	CBAM
Accuracy	91.48(90.12,91.85)	96.92(95.88,97.52)	98.17(97.54,98.96)
Precision	91.65(90.44,92.12)	95.78 (94.66,96.32)	98.54(97.55,99.31)
Recall	91.33(90.24,91.77)	94.13(93.55,95.20)	99.29(98.90,99.72)
F-score	91.06(89.89,91.65)	94.95(93.78,95.62)	98.91(97.96,99.41)
Param.(M)	1.85	2.33	2.45

**Table 9:** Time series module ablation comparison results.

Metric	None	LSTM	GRU
Accuracy	95.20 (94.00, 96.20)	97.80 (96.50, 98.20)	98.17(97.54,98.96)
Precision	94.44 (93.20, 95.20)	96.92 (96.10, 97.20)	98.54(97.55,99.31)
Recall	94.32 (93.00, 95.00)	97.33 (96.24, 98.00)	99.29(98.90,99.72)
F1-score	94.38(93.1,95.1)	97.12(96.17,97.6)	98.91 (98.22,99.51)
Param.(M)	1.85	2.33	2.45

As presented in Table 7, introducing data augmentation significantly improves the model’s performance across all metrics compared to the non-augmented baseline. Specifically, the F1-score increases from 93.15% (no augmentation) to 98.89% with SMOTE, and 98.22% with WGAN-based augmentation. This confirms that data augmentation is a critical strategy for mitigating class imbalance and enhancing classification accuracy in ECG signal analysis.

When comparing the two augmentation methods, SMOTE outperforms WGAN across all four evaluation metrics. SMOTE achieves the highest Accuracy (98.17%), Precision (98.54%), Recall (99.29%), and F1-score (98.89%), indicating not only better overall classification performance but also superior recognition of minority classes. In contrast, WGAN-based augmentation yields slightly lower Accuracy (97.28%) and Precision (97.67%), and although it improves Recall to 98.38%, it still falls short of SMOTE. This demonstrates that SMOTE offers a more consistent and robust performance improvement.

Moreover, WGAN-based augmentation generally involves a more complex and resource-intensive training process. Unlike SMOTE, which generates synthetic samples through deterministic interpolation, GANs require adversarial training and careful tuning of generator-discriminator dynamics. This often leads to longer training times and potential instability such as mode collapse. Prior studies (e.g., Ref. [45]) have also shown that WGAN-based methods demand significantly more computational resources and are less reliable when applied to small or highly imbalanced medical datasets.

Considering both empirical performance and implementation feasibility, SMOTE offers a better trade-off between effectiveness, computational cost, and ease of integration. Therefore, we adopt SMOTE as the final data augmentation strategy in this work.

In this experiment, we evaluated the impact of different attention mechanisms (no attention, SE, and CBAM) on the model’s performance. The experimental results are shown in Table 8. With fixed CNN and GRU conditions, the introduction of attention mechanisms significantly improved the model’s performance. Specifically, after incorporating the SE attention mechanism, the model’s accuracy increased to 97.32%, a notable improvement from the 94.02% accuracy achieved without attention mechanisms. Precision, recall, and F1-score also showed significant improvements. Further introduction of the CBAM mechanism resulted in even better performance across all metrics, with accuracy reaching 98.17%, precision at 98.54%, recall at 99.29%, and F1-score at 98.91%. These results indicate that attention mechanisms, particularly CBAM, can effectively enhance the model’s focus on important features, thereby achieving higher recognition performance in multi-class tasks.

In this experiment, we compared the impact of different time-series modeling modules (no time-series modeling, LSTM, and GRU) on the model’s performance. The experimental results are shown in Table 9. With fixed CNN conditions, different time-series modeling modules significantly affected the model’s performance metrics. First, after incorporating the LSTM module, the accuracy increased to 97.80%, an improvement over the 95.20% accuracy achieved without time-series modeling. Precision, recall, and F1-score also showed improvements, reaching 96.92%, 97.33%, and 97.12%, respectively. Further introduction of the GRU module resulted in an even greater performance boost, with accuracy reaching 98.17%, precision at 98.54%, recall at 99.29%, and F1-score at 98.91%. These results indicate that time-series modeling modules, especially GRU, can effectively capture the temporal information in the data, thereby significantly enhancing the model’s performance in multi-class tasks. Overall, the GRU module performed best across all metrics, confirming its effectiveness in time-series modeling.

In this experiment, we evaluated the contribution of different modules (CNN, CBAM, and GRU) to the model’s performance. The experimental results are shown in Table 10. First, when only using CNN, the model achieved an accuracy of 93.63%, precision of 93.77%, recall of 94.20%, and an F1 score of 93.98%. This indicates that the model’s performance is relatively limited when relying solely on CNN for feature extraction. Next, when the CBAM module was added, the model’s performance significantly improved, with accuracy increasing to 94.89% and the F1 score rising to 95.95%. By introducing the attention mechanism, CBAM effectively enhanced the model’s focus on important features, further improving classification performance.

After introducing the GRU module, the model’s performance was further optimized, reaching an accuracy of 96.88% and an F1 score of 95.73%. The GRU’s advantage in capturing temporal features allowed the model to perform better when handling ECG signals. Finally, when combining CNN, CBAM, and GRU, the model performed at its best, with accuracy reaching 98.17%, precision at 98.54%, recall at 99.29%, and an F1 score of 98.91%. This shows that combining CNN for feature extraction, CBAM for attention optimization, and GRU for temporal modeling effectively improves the overall performance of the model. In summary, the model combining CNN, CBAM, and GRU performs optimally in terms of accuracy and F1 score, confirming the important role of each module in the model architecture, especially in enhancing the performance of multi-class tasks.

## 5. Conclusions

This paper proposes an attention-enhanced CNN-GRU model, named CNN-CBAM-GRU, for intelligent diagnosis of cardiovascular diseases. By introducing the CBAM module, the model can adaptively adjust the importance of feature maps, thereby improving feature extraction and classification performance. The experimental results show that CNN-CBAM-GRU significantly outperforms the traditional CNN-GRU model in terms of accuracy in the 5-class classification task on the MIT-BIH arrhythmia database. Compared to other related works, this paper has several advantages. Many existing models focus on a single evaluation metric (such as accuracy) or fail to provide complete parameter information, which limits the comprehensiveness of the model evaluation. This paper not only conducts a comprehensive evaluation using multiple metrics (including accuracy, precision, recall, and F1-score), but also provides detailed information on the model’s parameter count (e.g., 2.45M parameters), offering a more comprehensive performance comparison. Additionally, the experiments include hyperparameter optimization, loss function analysis, performance comparison, and ablation studies, further validating the contribution of the CBAM module to model performance. Despite the significant results achieved, this study has certain limitations. For instance, the model’s computational complexity is relatively high, and practical applications may face challenges related to resource consumption. Future research can focus on optimizing the model’s computational efficiency to enable more efficient real-world applications. In summary, the CNN-CBAM-GRU model demonstrates significant performance improvements in intelligent cardiovascular disease diagnosis, providing an efficient and comprehensive solution for future research in this field, compared to traditional methods and models in existing literature.
